# Two Cases of Oculomotor Nerve Palsy in Idiopathic Intracranial Hypertension and a Literature Review

**DOI:** 10.1155/crnm/9730076

**Published:** 2026-05-11

**Authors:** Parth A. Patel, Jayachandra Muppa, Dan-Victor Giurgiutiu

**Affiliations:** ^1^ Department of Neurology, Medical College of Georgia, Augusta University, Augusta, Georgia, USA, augusta.edu

**Keywords:** case report, facial nerve, idiopathic intracranial hypertension, oculomotor nerve, ophthalmoparesis

## Abstract

Idiopathic intracranial hypertension (IIH) is characterized by elevated intracranial pressure in the absence of a secondary cause. Classically, it presents with headache, visual obscurations, pulsatile tinnitus, and diplopia, most commonly due to abducens nerve palsy. Involvement of other cranial nerves is rare. We report two patients with oculomotor nerve palsy in the setting of IIH. The first, a 26‐year‐old woman with a body mass index (BMI) of 35 kg/m^2^, had a unilateral complete oculomotor nerve palsy, with imaging supportive of IIH and lumbar puncture opening pressure > 55 cmH_2_O. The second, a 19‐year‐old man with a BMI of 38.5 kg/m^2^, had a unilateral partial oculomotor nerve palsy, bilateral abduction paresis, and left‐sided tongue dysgeusia, with imaging supportive of IIH and opening pressure of 36 cmH_2_O. Both demonstrated marked symptomatic improvement on acetazolamide. Including the patients initially presented here, a literature review identified 21 documented cases of oculomotor nerve palsy associated with apparent IIH. Of these, 13 satisfied contemporary or historic diagnostic criteria for IIH. These cases underscore the rarity of this manifestation and the importance of recognizing IIH as a potential cause of oculomotor nerve palsy to ensure timely diagnosis and management and to prevent irreversible vision loss.

## 1. Introduction

Idiopathic intracranial hypertension (IIH) is a condition characterized by elevated intracranial pressure (ICP) in the absence of an identifiable secondary cause, as defined by the 2013 Friedman criteria [1]. Despite geographic heterogeneity, the incidence of IIH is estimated to be approximately 0.5–2 per 100,000 in the general population, which rises to 12–20 per 100,000 in high‐risk groups, classically obese women of childbearing age [2]. Typical symptoms include headache, visual obscurations, pulsatile tinnitus, and diplopia, the latter of which usually results from abducens nerve palsy. Other cranial nerves are only rarely implicated [3]. Oculomotor nerve palsy is more commonly caused by intracranial aneurysms and ischemia, and its occurrence in the context of IIH is particularly unusual [4]. Indeed, only scattered case reports have documented this association [3]. Here, we describe two patients with oculomotor nerve palsy in the setting of IIH, one with isolated involvement and one with additional cranial nerve findings, and review the literature on this rare manifestation.

## 2. Case Reports

### 2.1. Case 1

A 26‐year‐old woman with a body mass index (BMI) of 35 kg/m^2^ and no other pertinent medical history presented to the hospital with a one‐week history of left eyelid ptosis, diplopia, pulsatile tinnitus, and intermittent bifrontal headaches. Examination revealed left eyelid ptosis; a mydriatic, nonreactive left pupil; and impaired adduction, elevation, and depression of the left eye. Visual acuity was 20/20 in the right eye and 20/100 in the left eye. Fundoscopy demonstrated bilateral papilledema and scattered hemorrhages in the right eye. No other focal neurologic deficits were appreciated.

**FIGURE 1:**
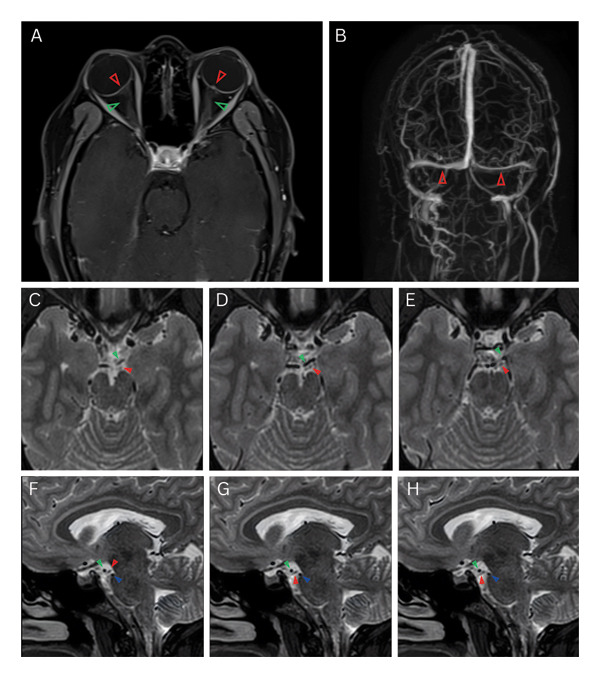
(A) Axial contrast‐enhanced T1‐weighted magnetic resonance imaging (MRI) of the orbits (with orbital fat suppression; 3‐mm slice thickness) revealing posterior globe flattening (red arrows) and tortuosity of the optic nerves (green arrows). (B) Magnetic resonance venography of the brain demonstrating bilateral transverse sinus stenosis (red arrows), with the left more stenotic than the right. (C–E) Axial T2‐weighted MRI of the brain (0.9‐mm slice thickness) delineating the course of the left oculomotor nerve (red arrow) under the left posterior cerebral artery (PCA; green arrow). (F–H) Sagittal T2‐weighted MRI of the brain (1‐mm slice thickness) showing the course of the left oculomotor nerve (red arrow) between the left PCA (green arrow) and the superior cerebellar artery (blue arrow).

Due to concern for a compressive etiology of symptoms, neuroimaging was emergently obtained. Contrast‐enhanced magnetic resonance imaging (MRI), computed tomography (CT), and CT angiography (CTA) of the brain revealed posterior globe flattening, tortuosity of the optic nerves, bilateral transverse sinus stenoses, and a partially empty sella, radiological findings supportive of IIH (Figure 1(A)). Contrast‐enhanced MRI of the orbits detected no focal pathology, while magnetic resonance venography (MRV) of the brain confirmed the presence of transverse sinus stenoses, with the left more stenotic than the right (Figure 1(B)). A diagnostic lumbar puncture showed an opening pressure greater than 55 cmH_2_O with normal CSF analysis. Serologic testing, including antinuclear antibody (ANA), antineutrophil cytoplasmic antibodies (ANCA), human immunodeficiency virus (HIV), and syphilis screen, was negative. Cerebral angiography, obtained on hospital day two, further excluded aneurysms or other vascular malformations as the cause of the palsy.

Taken together, the presence of papilledema, markedly elevated CSF opening pressure (> 55 cmH_2_O) with normal CSF composition, neuroimaging without structural or vascular pathology, and the exclusion of secondary causes fulfilled the diagnosis for IIH as defined by the 2013 Friedman criteria [1].

As such, acetazolamide 500 mg twice daily was initiated on hospital day one (treatment day zero). By treatment day two, some improvement in mydriasis was noted, though other ocular deficits persisted. She was discharged on treatment day three on the same dose, with weight loss counseling and a recommendation to initiate a glucagon‐like peptide‐1 (GLP‐1) receptor agonist in the outpatient setting. At 6‐week follow‐up, headaches and ocular symptoms had markedly improved, with resolution of ptosis and only mild left ophthalmoparesis. By 7 months, all symptoms had resolved, and she remained on a stable acetazolamide regimen. Given the potential for pregnancy, alternative management strategies for IIH, including transverse venous sinus stenting, were discussed with the patient, although further intervention was deferred by the patient.

### 2.2. Case 2

A 19‐year‐old man with a BMI of 38.5 kg/m^2^, but otherwise healthy, presented to the hospital with acute‐onset bitemporal headaches, left eyelid ptosis, and diplopia. He had initially sought care at an outside hospital for similar symptoms and was transferred for further assessment. CTA of the brain at the outside hospital showed no evidence of an aneurysm. A diagnostic lumbar puncture performed there demonstrated an opening pressure > 55 cmH_2_O (in the sitting position), with CSF studies, including an encephalitis/meningitis panel, that were within normal limits.

Upon admission to this institution, ocular examination revealed left eyelid ptosis; a mydriatic, reactive left pupil; impaired adduction, elevation, and depression of the left eye; and bilateral abduction paresis. Visual acuity was 20/20 in the right eye and 20/40 in the left. Fundoscopy showed bilateral papilledema. He further reported hypogeusia of the left side of the tongue. No other focal neurologic deficits or signs of meningeal irritation were appreciated.

**FIGURE 2:**
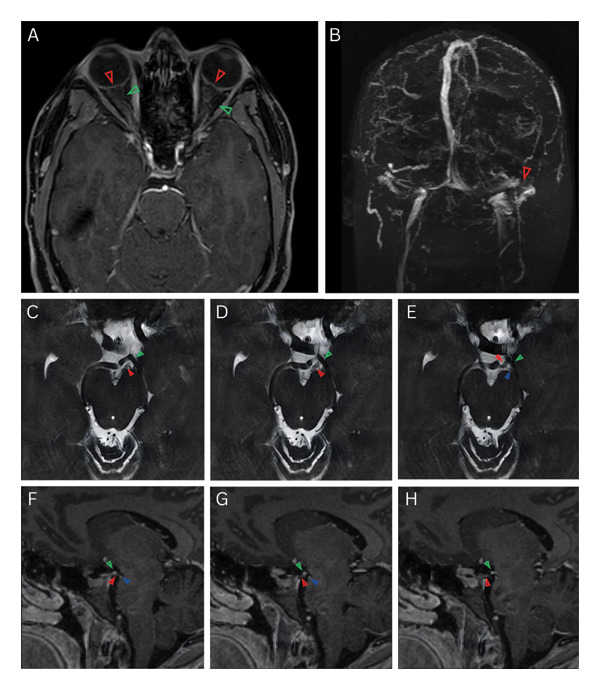
(A) Axial contrast‐enhanced T1‐weighted magnetic resonance imaging (MRI) of the orbits (with orbital fat suppression; 0.9‐mm slice thickness) demonstrating bilateral optic nerve head cupping (red arrows) and tortuosity of the optic nerves (green arrows), with left more tortuous than right. (B) Magnetic resonance venography of the brain revealing a left transverse sinus stenosis (red arrow). (C–E) Axial fast imaging employing steady‐state acquisition (FIESTA) MRI of the brain (1.4‐mm slice thickness) delineating the course of the left oculomotor nerve (red arrow) under the left posterior cerebral artery (PCA; green arrow) and above the left superior cerebellar artery (SCA; blue arrow). (F–H) Sagittal contrast‐enhanced T1‐weighted MRI of the brain (1‐mm slice thickness) showing the course of the left oculomotor nerve between the left PCA (green arrow) and SCA (blue arrow).

The constellation of multiple concomitant cranial nerve palsies preliminarily suggested either a structural or inflammatory etiology. Contrast‐enhanced MRI of the brain, face, neck, and orbits demonstrated bilateral optic nerve head cupping, tortuosity of the optic nerves, and distention of the optic nerve sheaths, without focal pathology (Figure 2(A)). MRV of the brain revealed left transverse‐sigmoid sinus dominance with associated stenosis of the distal left transverse sinus (Figure 2(B)). Repeat diagnostic lumbar puncture measured an opening pressure of 36 cmH_2_O (in the lateral recumbent position). CSF analysis, including cytology, demonstrated a mild pleocytosis of 7 cells/μL but was otherwise unremarkable. Serologic testing, including syphilis screen; HIV; hepatitis panel; serum protein electrophoresis; ANA; anti‐Smith, anti‐SSA, anti‐SSB, double‐stranded DNA, thyroid peroxidase, and ANCA; and Lyme serologies, was negative.

In the context of papilledema, elevated CSF opening pressure, supportive neuroimaging findings, and the exclusion of secondary causes, these findings largely satisfied the diagnostic criteria for IIH [1], noting the mild CSF pleocytosis as a minor departure from the requirement for normal CSF composition.

Accordingly, acetazolamide 500 mg twice daily was initiated on hospital day three (treatment day zero). On treatment day one, mild improvement in left eye ophthalmoparesis was noted. By treatment day two, ptosis and extraocular movement deficits had markedly improved, with near‐complete resolution of mydriasis. Persistent deficits prompted escalation of acetazolamide to 500 mg three times daily on treatment day four. He was discharged on treatment day six (hospital day nine) on that regimen, with substantial improvement in right eye abduction and continued recovery of left eye ocular motor function. Weight loss counseling was provided, with plans to taper acetazolamide after achieving a 10%–20% reduction in body weight. At 4‐month follow‐up, his ophthalmoparesis had resolved, while his left pupil remained slightly larger than the right. After achieving the targeted weight reduction, acetazolamide was gradually tapered over several months and completely discontinued by 1 year from treatment initiation. One year after treatment cessation, the patient had recurrent headache with papilledema, requiring reinitiation of acetazolamide 500 mg three times daily, which was titrated to four times daily due to persistent symptoms. Given recurrent episodes of nephrolithiasis, this medication was discontinued, and topiramate 100 mg twice daily and potassium citrate 45 mEq twice daily (for prevention of renal calculi) were initiated. Symptoms abated on this regimen.

## 3. Discussion

Despite the relative rarity of oculomotor nerve involvement in IIH, these cases highlight that it should be recognized as a possible neurological sequela of the condition. Both patients met the most recent diagnostic criteria for IIH [1], with extensive evaluation revealing no alternative etiology for the presenting findings. Apart from cranial nerve abnormalities, their neurological examinations were otherwise unremarkable. Contrast‐enhanced neuroimaging demonstrated no obstructive or structural lesions, although both patients exhibited transverse sinus stenosis, a finding that supports the diagnosis [5]. One patient had a mild CSF pleocytosis, which may have been reactive following a recent lumbar puncture [6] but does introduce some degree of diagnostic uncertainty. The discrepancy between the markedly elevated opening pressure (> 55 cmH_2_O) measured at the outside hospital and the subsequently lower measurement (36 cmH_2_O) at our institution may reflect patient positioning (sitting vs. lateral recumbent position), physiologic fluctuation in ICP, or partial pressure reduction following initial CSF removal.

Our literature review (see Supporting information [available here] for methodology) identified sporadic reports of oculomotor nerve palsy thought to be secondary to IIH (Table 1) [7–23, 25]. Diagnostic criteria for IIH have evolved substantially since their introduction [24, 26, 27], with the most recent iteration representing the strictest yet [1]. Of the 21 reported cases, only six entirely satisfied the 2013 Friedman criteria based on the information provided [12, 20, 22, 23]. Nevertheless, seven were classified as presumed IIH based on earlier definitions [24, 26, 27]. Cases were deemed not to be IIH if diagnostic criteria were not clearly delineated [8, 9, 17], if intracranial hypertension was not idiopathic, i.e., secondary to medication use or another medical condition [14, 15, 18], or if an alternative diagnosis was suspected [10, 17].

**TABLE 1:** Reported cases of oculomotor nerve palsy presumed secondary to idiopathic intracranial hypertension.

First author (year)	Age (years)	Sex	Cranial nerve findings	BMI (kg/m^2^)	CSF OP (cmH_2_O)	Imaging findings	Etiology/notes	Management for IIH
Sperry (1979) [7]	34	F	Right inferior rectus and medial rectus paresis; right pupil dilated and sluggishly reactive to light	Not specified	57	Contrast‐enhanced CT, carotid angiogram normal	Presumed IIH	Acetazolamide

Snyder (1979) [8]	25	F	Bilateral ophthalmoplegia and dilated pupils; right eye ptosis; left facial nerve palsy	48.5	> 30	Skull X‐ray normal; CT with small ventricles; normal cerebral angiogram	Presumed IIH	Dexamethasone

McCammon (1981) [9]	24	F	Complete left oculomotor nerve palsy	Obese; not specified	55	Skull X‐ray, CT, four‐vessel angiogram normal	IIH diagnostic criteria not formally defined	Prednisolone and acetazolamide

Landan (1987) [10]	28	F	Right RAPD; complete bilateral external ophthalmoplegia	Not specified	45	CT with slit‐like ventricles; cerebral angiography normal	IIH diagnostic criteria not formally defined	Dexamethasone, acetazolamide, and optic nerve sheath fenestration

Kidron (1989) [11]	18	F	Complete right ophthalmoplegia; global left ophthalmoparesis; bilateral dilated, nonreactive pupils; bilateral diminished corneal reflexes; bilateral facial palsy	Obese; not specified	> 60	CT, MRI normal; four‐vessel cerebral angiography with very slow vascular passage time	Upper respiratory infection 10 days prior; absent bilateral lower extremity reflexes	Dexamethasone, acetazolamide, furosemide, lumbar drain, and lumboperitoneal shunt
	26	F	Bilateral ophthalmoparesis; transient unilateral facial paresis	Obese; not specified	> 60	CT normal; four‐vessel cerebral angiography with slow vascular passage time	Marked lower limb hyporeflexia	Dexamethasone, acetazolamide, furosemide, and lumbar drain

Chansoria (2005) [12]	7	M	Left eye ptosis and medial rectus palsy; right lateral rectus palsy	Not specified	26	CT/MRI normal	Presumed IIH	Acetazolamide

Bruce (2006) [13]	19	F	Bilateral horizontal and vertical eye movement impairment in all directions	Obese; not specified	63	Contrast‐enhanced MRI normal; MRV normal	IIH	Acetazolamide and lumboperitoneal shunt

Thapa (2008) [14]	5.5	F	Bilateral ptosis and medial rectus palsy	Normal weight; not specified	28	MRI normal	Presumed IIH	Furosemide and acetazolamide

Tan (2010) [15]	14	F	Bilateral mild ptosis and medial and inferior rectus palsy	Normal weight; not specified	35	Contrast‐enhanced MRI normal; MRV normal	Secondary; suspected due to acute maxillary sinusitis	Acetazolamide

Rezazadeh (2010) [16]	21	F	Complete right oculomotor nerve palsy	Not specified	40	CT/CTA/MRI/MRV normal	Secondary; isotretinoin‐associated	Acetazolamide

Wani (2015) [17]	22	F	Bilateral ptosis and dilated, nonreactive pupils; Complete right ophthalmoplegia; left abducens nerve palsy	25	30	MRI with tortuous bilateral optic nerves, increased perineural CSF spaces, mild flattening of bilateral posterior globe of sclera; MRV normal	Presumed IIH	Acetazolamide, topiramate, and unspecified diuretics

Ragab (2017) [18]	17	F	Bilateral dilated, nonreactive pupils; right eye limited adduction; bilateral abduction deficits	Overweight; not specified	> 50	MRI with tortuous optic nerve, flattened posterior eye globes, slit‐like ventricles; MRV with attenuation of both transverse sinuses, left greater than right	Hypotonia and quadriparesis in all extremities; absent reflexes; IIH diagnostic criteria not formally defined	Acetazolamide and lumboperitoneal shunt

Wright (2017) [19]	16	F	Left eye ptosis; bilateral adduction deficits, impaired left downgaze; right pupil 1 mm larger than left, no RAPD	Not specified	> 100	MRI/MRV normal	Secondary; minocycline‐associated	Discontinued minocycline and initiated acetazolamide

Karimi (2020) [20]	25	F	Complete left third nerve palsy	Not specified	28	MRI with empty sella, bilateral optic nerve hydrops and tortuosity, flattening of posterior aspects of globes; MRV with bilateral transverse sinus stenosis	Presumed IIH	Topiramate

Interlandi (2020) [21]	16	F	Bilateral complete ophthalmoplegia; bilateral pupils 5 mm and sluggishly reactive to light, no RAPD	27	88	Contrast‐enhanced MRI with optic nerve sheath distention, globe flattening with optic nerve head protrusion, empty sella; MRV normal	IIH	External lumbar drainage and lumboperitoneal shunt

Mathkour (2021) [22]	26	F	Right eye ptosis, complete ophthalmoplegia; bilateral mydriasis, right pupil sluggishly reactive to light	> 35	55	CT/MRI normal; MRV, dural sinus venography with sinus stenosis	Presumed IIH	Acetazolamide, furosemide, and venous sinus stenting

Hesham (2024) [23]	29	F	Complete right oculomotor palsy; bilateral limited abduction	36	> 55	CT/CTA/CTV normal; contrast‐enhanced MRI with bilateral posterior scleral flattening	IIH	Acetazolamide and ventriculoperitoneal shunt

Vellingiri (2025) [24]	50s	F	Right eye ptosis and medial rectus palsy	Not specified	26	Contrast‐enhanced CT normal; MRI/MRA/MRV showed bilateral posterior scleral flattening and bilateral optic nerve tortuosity	IIH	Acetazolamide and topiramate

Present case one	26	F	Complete left oculomotor palsy	35	> 55	Contrast‐enhanced CT/CTA/MRI with posterior globe flattening, bilateral optic nerve tortuosity, transverse sinus stenoses; MRV confirmed stenoses; cerebral angiogram normal	IIH	Acetazolamide

Present case two	19	M	Left eye ptosis, dilated, reactive pupil, complete ophthalmoparesis; right abduction paresis; hypogeusia of left tongue	38.5	36	CTA normal; contrast‐enhanced MRI of the brain, face, neck, and orbits with bilateral optic nerve head cupping, optic nerve sheath distention; MRV with left transverse sinus stenosis	IIH	Acetazolamide

*Note:* CSF, cerebrospinal fluid; F, female; M, male.

Abbreviations: BMI, body mass index; CT, computed tomography; CTA, computed tomography angiography; IIH, idiopathic intracranial hypertension; MRA, magnetic resonance angiography; MRI, magnetic resonance imaging; MRV, magnetic resonance venography; RAPD, relative afferent pupillary defect.

Consistent with broader epidemiological patterns of IIH [2], most reported cases were in young (average age 21.1 years), obese (average BMI 35 kg/m^2^; data available for six patients) women (85%). Most oculomotor nerve palsies were described as unilateral (69%) [11, 16, 19, 21–23, 25] and partial (62%) [11–13, 21–23, 25], though several were bilateral (31%) [7, 12, 13, 20] and complete (38%) [7, 16, 19, 22]. Pupillary involvement was explicitly documented in eight patients [16, 19–22, 25]. Notably, opening pressures were frequently markedly elevated, ranging from 26 to over 100 cmH_2_O. As illustrated by our second case, concurrent involvement of other cranial nerves, including trochlear (38%) [7, 16, 20, 21], abducens (62%) [7, 11, 12, 16, 20–22], and facial (15%) [7], may also occur. Such broader cranial nerve involvement should prompt consideration of other processes affecting the cavernous sinus, skull base, or brainstem, including inflammatory conditions (e.g., neurosarcoidosis), neoplastic infiltration, infections (e.g., tuberculous meningitis), or vascular lesions (cavernous sinus thrombosis) [28]. However, when appropriate evaluation reveals no alternative etiology, IIH remains a plausible explanation.

Multiple cranial nerve palsies have been reported in association with IIH, with varying degrees of prevalence. Abducens nerve palsy occurs in approximately 14% of patients with IIH and represents the most ubiquitous nonvisual neurologic deficit associated with the condition [29]. Its frequency is sufficient that it is considered to be a supportive diagnostic feature in clinical guidelines [1]. Trochlear nerve involvement is less well documented, with only isolated case reports and small series describing vertical diplopia or hypertropia attributable to trochlear nerve palsy in the context of IIH [3, 21]. Similarly, the trigeminal nerve may be affected, presenting as unilateral sensory loss or neuralgia [3, 30]. Facial nerve involvement usually manifests as mild, transient lower motor neuron facial weakness [3, 31], though, as in our second case, lingual hypogeusia may occur. Its reported prevalence in the literature ranges from 2% to 6% [32]. Finally, although very uncommon, lower cranial nerves, including the glossopharyngeal, vagus, and hypoglossal nerves, have been implicated, usually in the context of severe disease [3].

Abducens nerve palsy is a classic false localizing sign of elevated ICP. Its susceptibility to injury has traditionally been attributed to its protracted intracranial course, during which it may be stretched or compressed against the petrous ligament or ridge [33]. Alternatively, Harvey Cushing proposed that downward CSF pulsations in the setting of elevated ICP may strangulate the nerve against the anterior inferior cerebellar artery (or other lateral branches of the basilar artery), producing grooving of the nerve at its pontine exit [34]. In contrast, the mechanism of oculomotor nerve palsy in IIH remains less well defined. After exiting the midbrain, the oculomotor nerve traverses the subarachnoid space between the posterior cerebral artery and superior cerebellar artery (Figures 1(C), 1(D), 1(E), 1(F), 1(G), 1(H), 2(C), 2(D), 2(E), 2(F), 2(G), and 2(H)) and briefly courses alongside the posterior communicating artery [28]. Compression here, as observed with intracranial aneurysms, may produce an oculomotor nerve palsy. It is conceivable that a mechanism analogous to that proposed by Cushing for abducens nerve palsy, namely vascular compression exacerbated by downward CSF pulsations [34], could contribute to oculomotor nerve dysfunction in the setting of markedly elevated ICP. Additional vulnerability exists after the nerve enters the cavernous sinus, where increased pressure may concomitantly implicate the trochlear and abducens nerves as well as the first and second divisions of the trigeminal nerve [21]. Other proposed theories include impaired perfusion or altered CSF dynamics in the posterior fossa [3, 21]. Considering the limited number of documented cases, it remains unclear whether a single mechanism underlies oculomotor involvement in IIH. Variability in pupillary involvement and laterality among reported patients certainly suggests multiple potential sites of vulnerability [3].

Irrespective of the precise mechanism underlying oculomotor nerve palsy in IIH, prompt treatment is warranted to preserve neurologic and visual function. Of the 13 patients with IIH we reviewed, four ultimately required procedural intervention for definitive management [12, 20–22]. The remainder were managed medically.

In conclusion, oculomotor nerve palsy is a rare but recognized manifestation of IIH that can mimic more common etiologies such as an aneurysm or ischemia. Clinicians should be cognizant of this association to ensure timely and accurate diagnosis and to prevent irreversible vision loss. Reporting additional cases may help further clarify underlying mechanisms and clinical patterns.

## 4. Patient Perspective

### 4.1. Patient One

“I started having problems moving my left eye. I noticed my left eye was drooping in the mirror. I was also having a headache that would come and go. My symptoms did not improve after a week, so I decided to come into the emergency department. After I received the spinal tap the whooshing noise in my ear went away and my headache improved. After I started the medication, my eye felt better, but it took a few months for my symptoms to completely go away. Now my symptoms are gone.”

### 4.2. Patient Two

“One of the first symptoms that cropped up was light sensitivity, not too dissimilar to that of a migraine (so I’ve heard) or a hangover. A persistent broad aching of the whole of my head came around the same time, which you can imagine was bothersome to say the least, but at this point, it was livable. I later acquired a distinct alteration to my sense of taste, making food quite unpalatable and just general discomfort that made sleeping well an out‐of‐reach fantasy, despite my efforts for the two stubborn weeks before I relinquished and went to the ER. Another fun symptom that definitely lasted the longest was this rather odd effect of my left eye losing its ability to move or do much of anything really. One of the later symptoms to arrive, and I think the last one to leave by a wide margin, still being annoying for a month or so after my discharge. ”

“I would say that as far as the effectiveness of treatment goes, the first medication I received worked well, I think. A couple bonuses from this first medication were that as my body was growing accustomed to it, my extremities would tingle as if mildly hyperventilating, and I passed no fewer than 3 different kidney stones, one of which was in hospital sometime later. Another medication I was switched to was more convenient as it was less pills, no kidney stones, and generally was a better time.”

## Author Contributions

P.A.P., J.M., and D‐V.G. contributed to the conception and design of the article, the drafting of the paper, and revising it critically for intellectual content.

## Funding

The authors have nothing to report.

## Disclosure

All authors provided final approval of the version to be published and agree to be accountable for all aspects of the work.

## Ethics Statement

Informed consent was obtained from both patients. IRB approval was not required, and ethical guidelines as outlined in the Declaration of Helsinki were followed.

## Conflicts of Interest

The authors declare no conflicts of interest.

## Supporting Information

Additional supporting information can be found online in the Supporting Information section.

## Supporting information


**Supporting Information** File contains complete methodology for literature review.

## Data Availability

Data sharing is not applicable to this article as no datasets were generated or analyzed during the current study.
