# Supplementary material for: Two Cases of Oculomotor Nerve Palsy in Idiopathic Intracranial Hypertension and a Literature Review
Source: Case Rep Neurol Med. 2026 May 11;2026:9730076. doi: 10.1155/crnm/9730076 (PMC13159091; doi:10.1155/crnm/9730076)
Supplement: Supplementary file 1 — Supporting Information File contains complete methodology for literature review. [file CRNM-2026-9730076-s001.docx]

**Supplemental Materials**

On October 2025, the PubMed database was queried using the terms ("Oculomotor Nerve Diseases" OR "Ophthalmoparesis" OR "Ophthalmoplegia" OR "oculomotor nerve palsy" OR "cranial nerve III palsy" OR "third nerve palsy" OR "oculomotor palsy") AND ("Idiopathic Intracranial Hypertension" OR "pseudotumor cerebri" OR "benign intracranial hypertension" OR "IIH"), which yielded 53 results. Two authors (PAP, JM) examined each abstract to identify cases describing cranial nerve III palsy in the setting of apparent idiopathic intracranial hypertension (IIH). Articles were excluded if they did not explicitly report cranial nerve III palsy or provide sufficient clinical detail to confirm its presence alongside a reported diagnosis of IIH. The remaining articles and their references were comprehensively reviewed, and 18 relevant articles were included in this review (**Table 1**).
